# Characteristics and Outcomes of mHealth Interventions in Psychosis: Systematic Mapping Review

**DOI:** 10.2196/55924

**Published:** 2024-12-23

**Authors:** Pei Yi Loh, Laura Martinengo, Creighton Heaukulani, Xin Yang Tan, Moses Hng, Yong Yin Cheah, Robert J T Morris, Lorainne Tudor Car, Jimmy Lee

**Affiliations:** 1 Lee Kong Chian School of Medicine Nanyang Technological University Singapore Singapore; 2 Centre for Behavioural and Implementation Sciences Interventions Yong Loo Lin School of Medicine National University of Singapore Singapore Singapore; 3 Office for Healthcare Transformation Ministry of Health Singapore Singapore; 4 Institute of Mental Health Singapore Singapore; 5 Yong Loo Lin School of Medicine National University of Singapore Singapore Singapore; 6 Department of Primary Care and Public Health School of Public Health Imperial College London London United Kingdom

**Keywords:** mHealth, digital health, mobile applications, psychosis, schizophrenia, schizophrenia spectrum, psychotic disorders, mapping review

## Abstract

**Background:**

Mobile health (mHealth) interventions have gained popularity in augmenting psychiatric care for adults with psychosis. Interest has grown in leveraging mHealth to empower individuals living with severe mental illness and extend continuity of care beyond the hospital to the community. However, reported outcomes have been mixed, likely attributed in part to the intervention and adopted outcomes, which affected between-study comparisons.

**Objective:**

This study aimed to critically review outcome measures used to evaluate mHealth interventions for adults with psychosis in relation to the characteristics of mHealth interventions.

**Methods:**

A systematic mapping review was conducted. We searched PubMed, CINAHL, Embase, PsycINFO, and Cochrane Libraries from 1973 to the present. Selection criteria included randomized controlled studies of mHealth interventions in adults diagnosed with schizophrenia spectrum disorders. Reviewers worked in pairs to screen and extract data from included studies independently using a standardized form; disagreements were resolved by consensus with an independent reviewer. We report our findings in line with PRISMA-ScR (Preferred Reporting Items for Systematic Reviews and Meta-Analyses extension for Scoping Reviews) guidelines.

**Results:**

A total of 1703 citations were screened; 29 publications reporting on 23 studies were included in this review. mHealth interventions for psychosis span a wide range, with psychological therapy being the most-deployed intervention (12/23, 52%), followed by psychoeducation (8/23, 35%) and active self-monitoring (8/23, 35%). Several mHealth interventions for psychosis targeted multiple pillars of biopsychosocial well-being (10/23, 43%); the bulk of interventions (16/23, 70%) incorporated features promoting users’ self-management. The majority of mHealth interventions were delivered through applications (14/23, 61%) as the main medium and smartphones (17/23, 74%) as the main channel of delivery. Interventions were primarily administered in the outpatient and community settings (16/23, 70%); many were also blended with in-person sessions (11/23, 48%) or guided remotely (6/23, 26%) by persons, including health care providers or trained peer supporters. The severity of psychosis-related symptoms (21/23, 91%) was the most prevalent outcome, of which positive symptoms (13/23, 57%), mood and anxiety (10/23, 43%), and overall psychopathology severity (9/23, 39%) were most commonly measured. Patient-centric outcomes, including well-being (17/23, 74%)—particularly quality of life (10/23, 43%)—and user experience (15/23, 65%), including feasibility (7/23, 30%), acceptability (7/23, 30%), and engagement (7/23, 26%). Notably, outcome choices remained diverse despite stratification by type of mHealth intervention.

**Conclusions:**

mHealth interventions for psychosis encompass a wide range of modalities and use outcome measures that probe various social and behavioral determinants of health. These should be considered complex interventions, and a holistic evaluation approach combining clinical and patient-centric outcomes is recommended.

## Introduction

Psychotic disorders are a group of serious mental illnesses consisting of “abnormalities in one or more of the following five domains: delusions, hallucinations, disorganized thinking (speech), grossly disorganized or abnormal motor behavior (including catatonia), and negative symptoms” [1]. The global burden of psychotic disorders on individuals, communities, and health care systems cannot be understated. Schizophrenia, the most well-known psychotic disorder, has been ranked among the top 20 causes of years lived with disability (YLDs) among all illnesses and injuries and afflicts 23.6 million individuals worldwide [2]. Schizophrenia also has an early age of onset in adolescence [3] and has a propensity for relapsing. Such acute relapses can leave deleterious impacts on the individual yet are difficult to predict. The chronic, relapsing-remitting illness trajectory of schizophrenia not only contributes to a high disability weight during acute psychotic episodes [4] but also incurs hefty societal costs—upwards of US $300 billion in the United States alone [5].

The debilitating nature of psychosis thus spells the need for scalable, cost-effective, and accessible solutions to augment traditional psychiatric management, which remains underpinned by intensive human-delivered care. Mobile health (mHealth), defined as the use of wireless mobile technologies for public health [6], stands out as an emerging possibility. This is especially so with smartphone ownership becoming more pervasive in the 21st century, including among persons living with psychotic disorders [7]. In an era where different smart devices permeate everyday life, mHealth can enable data-driven assessment of individuals’ lifestyles and well-being. The repertoire of personalized mHealth interventions has similarly been expanding, ranging from medication adherence tools [8] to internet-based cognitive behavioral therapy (iCBT) [9]. mHealth interventions, therefore, harbor much optimism in empowering persons living with psychosis toward proactive self-care with timely symptom management and targeted interventions [10].

With the exponential increase in popularity of mHealth in recent years [11], there is substantial interest in evaluating the efficacy of mHealth interventions for adult patients with psychosis. However, to the best of our knowledge, there are no systematic reviews evaluating mHealth interventions for adult patients with psychosis. A systematic meta-review of mHealth interventions for mental health in general [12] found that none of the included meta-analyses studied their effects on psychotic disorders. There have been a few reviews looking into mHealth technologies for psychosis, but these focused on the scope of technologies rather than the outcomes these technologies seek to achieve [13-15]. Reviews attempting to quantify the impact of mHealth interventions on psychosis outcomes included few articles at the time of publication [16], which may no longer represent the current body of mHealth interventions. Furthermore, Firth and Torous [17] assessed the impact of mHealth intervention only for feasibility, while Clarke et al [18] focused on their effectiveness in reducing psychotic symptoms without assessing other patient-centric outcomes.

It is therefore important to critically review prevailing outcome measures used to evaluate mHealth interventions for adults with psychosis, whether in terms of traditional clinical outcomes (eg, relapse prevention, reduction of hospitalization, or mortality) or person-centric attributes (eg, quality of life, subjective well-being, and various psychological constructs). This can be achieved through a systematic mapping review to “collate, describe and catalogue available evidence” while following “the same rigorous, objective and transparent processes as do systematic reviews” [19]. Although systematic reviews of randomized controlled trials (RCTs) are the perceived “gold standard,” there are significant hurdles in doing so, given the heterogeneity of mHealth interventions and study designs and lack of agreement over choices of outcomes and their measurement instruments. Against this backdrop, this systematic mapping review seeks to answer the following questions: (1) What are the characteristics of mHealth interventions for adults with psychosis? (2) What type of outcomes are assessed and reported in RCTs of mHealth interventions for adults with psychosis?

## Methods

### Overview

This mapping review was performed according to the methodology proposed by James et al [19] and reported in line with the PRISMA-ScR (Preferred Reporting Items for Systematic Reviews and Meta-Analyses extension for Scoping Reviews) reporting guidelines (Multimedia Appendix 1). The protocol was registered in Open Science Framework Registries [20] in May 2023.

### Setting Inclusion Criteria for Studies

Studies included in this mapping review were randomized controlled studies of any design, reporting the use of mHealth interventions for adults with psychosis. Our detailed inclusion and exclusion criteria are reflected in Textbox 1.

**Inclusion and exclusion criteria for randomized controlled trials (RCTs) reporting psychosis-focused mobile health (mHealth) interventions.**

Inclusion criteria:

- Article type:
  RCTs.
  Cluster RCTs.
  Quasi-RCTs.
  Randomized controlled feasibility studies.
- Language: English-language studies.
- Population:
  Adults above 18 years old diagnosed with schizophrenia spectrum disorders.
  Any inpatient, outpatient, or community setting.
  Any gender, ethnicity, or cultural background.
- Intervention:
  Any kind of mHealth intervention that is intended to alter, manage, or prevent changes in a patient’s behavior, emotions, cognition, functioning, or well-being.
  Examples of possible intervention types include delivering psychological therapy, psychoeducation, emergency assistance, self-monitoring, personalized recommendations on coping strategies, or medication adherence.
- Comparison: Studies with any type of control, such as:
  Another mHealth intervention.
  A non-mHealth intervention.
  Treatment-as-usual.
  A sham comparison or placebo.
  No intervention.
- Outcomes: All studies that evaluate the effectiveness of the mHealth intervention in terms of patient-related outcomes in psychosis, whether in terms of objective measures or patient-reported data.

Exclusion criteria:

- Article type:
  All other study designs, such as qualitative studies, review articles (including meta-analyses and scoping, literature, and systematic reviews), commentaries, editorials, opinion pieces, protocols, and observational studies (including cross-sectional studies, cohort studies).
  Conference abstracts, proceedings, and letters will be excluded unless a control group is present and there is sufficient data for extraction.
- Language: non–English language studies.
- Population:
  Organic psychosis, substance-induced psychosis, psychosis secondary to other medical conditions, and postnatal psychosis.
  Individuals with ultra-high risk (UHR) of psychosis.
  Caregivers or next-of-kin of patients and health care professionals.
- Intervention:
  Interventions that do not involve mHealth.
  Interventions that exclusively collect passive data.
  Interventions that only contain teleconferencing or virtual reality.
- Comparison: Single-arm interventional studies without a relevant control.
- Outcomes: Studies that exclusively assessed user experience-related or technical outcomes.

### Searching for Evidence

An electronic literature search was performed on September 20, 2022, across PubMed, CINAHL (EBSCO), PsycINFO (EBSCO), Embase (Ovid), and Cochrane Library. We included English language reports published from January 1973 onwards, aligning with the advent of mobile technologies. The search strategy was developed in PubMed and adapted to other databases in consultation with a medical librarian. Search terms included a comprehensive list of words and phrases representing the intersection between mHealth interventions and psychosis (Multimedia Appendix 2). During the screening process, the citations of reports identified from bibliographical databases were also searched to include any other eligible publications that met all the inclusion criteria.

### Screening Evidence

The search results from all databases were imported into a single EndNote (version 20; Clarivate) library, and duplicate records were removed. Subsequently, reviewers worked in pairs to select studies independently and in parallel using the online screening tool Covidence. This was performed in 2 stages: initial screening of title and abstract, followed by a second round of full-text screening. Discrepancies in any screening stage were resolved through a stepwise approach of mutual discussion, followed by engaging a third reviewer. The search and screening process was documented in a study selection flowchart [21].

### Coding

Data were coded using a standardized data extraction form on Microsoft Excel developed by the review team. Variables that were extracted included the following: study methodology, participant baseline characteristics, study setting, characteristics of mHealth intervention and delivery, choice of outcomes, and corresponding measurement instruments. The data extraction form was piloted in 2 studies and amended based on feedback before it was used for data extraction. Furthermore, reviewers met up regularly to ensure concordance in the data extraction process. Like the screening stage, reviewers worked in pairs to extract data from included studies independently and in parallel. The extracted data was compared, and any discrepancies were resolved through mutual discussion or involving a third reviewer acting as the arbiter.

### Describing and Visualizing the Findings

Data were visualized in a diagrammatic or tabular form accompanied by a narrative summary. Descriptive statistics were used to ascertain the choice of outcomes and measurement instruments reported in the included studies. Furthermore, data were classified and mapped according to the delivery medium (the digital platform used, eg, app, website, SMS), delivery channel (the physical device used, eg, smartphone, tablet), and delivery format. Based on recommendations by Lattie et al for digital mental health interventions, we modified their approach to subclassify delivery formats of mHealth interventions within this study based on the level of human support incorporated. Whereas self-guided interventions were defined to be fully automated, we consider guided interventions to “include human support as part of their delivery” (whether dyssynchronous or synchronous), while blended interventions involve the delivery of the digital modules “as part of face-to-face mental health interventions” [22].

Outcomes were grouped into 8 categories, namely severity of psychosis-related symptoms, functioning, well-being, medication adherence, adverse events, user experience, technical, and all other outcomes reported. These were derived directly or indirectly based on the International Consortium for Health Outcomes Measurement (ICHOM) patient-centered outcome measures for psychosis (eg, symptoms and functioning) [23], as well as the Core Outcome Measures in Effectiveness Trials (COMET) Initiative’s 38-item medical research outcome taxonomy (eg, 28: Emotional functioning/well-being, 32: Delivery of care, 38: Adverse events/effects) [24]. Instruments measuring any of these outcomes were also classified as objective or subjective, in line with the COSMIN (Consensus-based Standards for the selection of health Measurement INstruments) definition. [25] Objective measures include data passively obtained from phone usage or embedded sensors. Subjective measurement instruments include either observer-rated or self-report questionnaires that can be written or administered digitally, whether regarding their health status or experience using the mHealth intervention.

## Results

### Overview of Search Strategy

**Figure 1:**
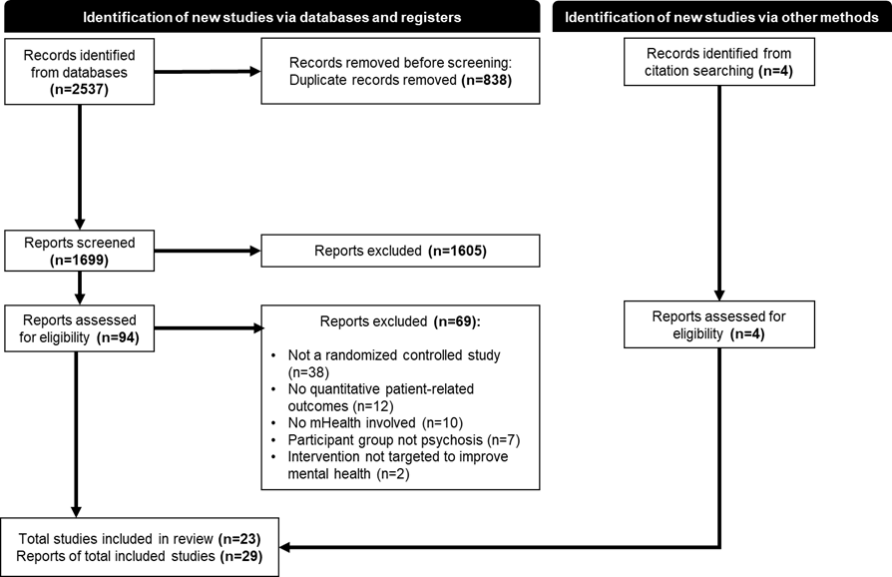
PRISMA flow diagram of study selection.

The initial search of databases yielded 2537 papers, of which a total of 1699 titles and abstracts were screened after removing duplicate records. Thereafter, 98 reports were assessed in full for eligibility, including 4 additional records, which were retrieved by searching the reference list of papers screened. This culminated in 29 publications reporting 23 studies being included in this review (PRISMA flow diagram in Figure 1).

### Characteristics of Included Studies

All included studies were published from 2017 onwards. Of these, nearly three-quarters of the included studies (17/23, 74%) were published from 2020 onwards [26-44]. Based on the 2021 World Bank Country and Landing Groups classification system, all except 2 studies [36,43] were conducted in high-income countries; 8/23 (35%) of the studies were conducted in the United States [32,38,44-52] and another 10/23 (43%) of the studies (including multicenter studies) were conducted in Europe [26,28,29,33-35,37,39-41,53,54]. The majority of the studies (16/23, 70%) were conducted in the outpatient and community setting [26,27,30-33,35-37,39-42,45-49,53,54]. Study designs included mostly RCTs with a variety of comparisons used. Particularly, two of the studies were 3-arm RCTs that featured both positive and negative controls [41,45].

There was also a mix of psychiatric diagnoses among recruited participants of the included studies. While over half (13/23, 57%) of included studies recruited only participants with psychotic disorders [26,28-32,34,36,39,40,42-44,47-49,52,53], 6 studies also recruited individuals with mood disorders (such as bipolar disorder or major depressive disorder) [27,33,38,45,46,50,51] and 1 study [35] included individuals with “Ultra-High Risk” of psychosis. By the American Psychiatric Association’s definition of adulthood, the mean participant age of most studies (16/23, 70%) 26-34,38,41-50,52] corresponded to middle adulthood (35 years and older) while that of remaining studies corresponded to young adulthood (35 years and younger). Table 1 presents a summary of the characteristics of the included studies, and further details can be found in Multimedia Appendix 3.

**Table 1:** Characteristics of included randomized controlled studies (N=23) reporting the use of mobile health (mHealth) interventions in psychosis.

Study characteristics			Studies, n (%)
**Year of latest publication**			
	Before 2020	6 (26)	
	2020 or after	17 (74)	
**Country**			
	United States	8 (35)	
	United Kingdom	4 (17)	
	Australia	1 (4)	
	China	1 (4)	
	Denmark	1 (4)	
	France	1 (4)	
	Poland	1 (4)	
	South Korea	1 (4)	
	Netherlands	1 (4)	
	More than 1 country	4 (17)	
**Setting**			
	Outpatient and community	16 (70)	
	Inpatient	1 (4)	
	Not specified	6 (26)	
**Study design**			
	Randomized controlled trial (RCT)	20 (87)	
	Quasi-RCT	2 (9)	
	Cluster RCT	1 (4)	
**Type of control**			
	Treatment as usual (TAU)	9 (39)	
	Another mHealth intervention	3 (13)	
	Different version of mHealth intervention	3 (13)	
	Waitlist control	3 (13)	
	Non-mHealth intervention	2 (9)	
	Placebo	1 (4)	
	TAU + another mHealth intervention	1 (4)	
	TAU + non-mHealth intervention	1 (4)	
**Type of participant diagnosis**			
	Psychotic disorders only	13 (57)	
	Psychotic disorders + mood disorders	6 (26)	
	Ultra-High Risk + psychotic disorders	1 (4)	
	Not specified	3 (13)	
**Participant mean age**			
	<35	7 (30)	
	35 and older	16 (70)	

### Characteristics of mHealth Interventions

A wide range of features were incorporated into the mHealth interventions investigated in included studies, spanning from psychological therapy (12/23, 52%) [26,28,29,32,38,40,43,45-49,52-54], psychoeducation (8/23, 35%) [32,36,38,40,42,45,50-53], active self-monitoring (8/23, 35%) [27,30,31,33-35,37,43,50,51], medication adherence (7/23, 30%) [33,34,36,40,41,44,45,53], personalized recommendations on coping strategies (5/23, 22%) [27,30,31,33-35] to peer support (3/23, 13%) [32,43,52]. Different types of psychological therapy were used, such as cognitive behavioral therapy (CBT) [26,28,29,32,45,46,52,54], cognitive training [38,40,53], social cognition training [43], or a mix of techniques [47-49]. Less frequently incorporated features included emergency assistance [33], behavioral activation and mindfulness [52], shared decision-making [39], and teleconsultations [40,53].

To further characterize the type of mHealth interventions, these features were, in turn, grouped into distinct themes based on the pillars of Engel’s biopsychosocial model [55]: medication-related features (eg, medication reminders, teleconsultations) representing the biological aspect, psychological therapy, and social support (eg, peer support, emergency assistance). A fourth intervention type—self-management—was also identified, encompassing psychoeducation, active self-monitoring and personalized recommendations on coping strategies. This was found to be a highly prevalent modality targeted by mHealth interventions (16/23, 70%) [27,30-38,40,42,43,45,50-53]. Many mHealth interventions were found to be multimodal, combining features targeting multiple pillars of Engel’s biopsychosocial model (10/23, 43%) [32-34,36,38,40,43,45,52,53]. Even among unimodal interventions, a combination of multiple features was commonly used, such as active symptom monitoring with correspondingly tailored coping strategies [27,34,35], or medication reminders along with teleconsultations [40,53]. Of these, there was an even spread between self-management, psychological therapy, and medication or treatment-related interventions without any interventions that exclusively delivered social support.

The majority of mHealth interventions were delivered through applications (14/23, 61%) as the main medium [27,30,31,34,35,37-42,46,50-54], and smartphones (17/23, 74%) as the main channel of delivery [27,30,31,34-45,50-54]. Most of the interventions were also blended with in-person sessions (11/23, 48%) [26,27,32,33,35,39,40,42-45,53] or guided remotely (6/23, 26%) [28-31,36,37,50-52] by persons such as health care providers or trained peer supporters (Table 2).

**Table 2:** Characteristics of mobile health (mHealth) interventions for psychosis from included studies (n=23).

mHealth intervention characteristics				Studies, n (%)
**Intervention type**				
	Self-management only			6 (26)
	Psychological therapy only			5 (22)
	Medication-related only			2 (9)
	Social support only			0 (0)
	More than 1 type			10 (43)
**Features involved^a^**				
	**Psychological therapy**			12 (52)
		Cognitive behavioral therapy	7 (30)	
		Cognitive training	2 (9)	
		Social cognition training	1 (4)	
		Social cognition + cognitive training	1 (4)	
	Psychoeducation			8 (35)
	Active self-monitoring			8 (35)
	Medication adherence			7 (30)
	Personalized recommendations on coping strategies			5 (22)
	Peer support			
	Emergency assistance			3 (13)
	Behavioral activation and mindfulness			1 (4)
	Shared decision making			1 (4)
	Teleconsultations			1 (4)
**Delivery medium**				
	Application			14 (61)
	SMS or messaging applications			2 (9)
	Website			2 (9)
	More than 1 delivery medium			4 (17)
	Not specified			1 (4)
**Delivery channel**				
	Smartphone			17 (74)
	Mobile phone			1 (4)
	Tablet			1 (4)
	Smartphone + computer			3 (13)
	Tablet + computer			1 (4)
**Format of delivery**				
	Blended			11 (48)
	Guided			6 (26)
	Self-guided			4 (17)
	Not specified			2 (9)

^a^For this category, we note that the total sum of percentages will exceed 100% as some interventions come with multiple features.

### Outcome Measures

The most common primary outcome was the severity of psychosis-related symptoms, which was chosen in 11/23 (48%) of included studies [26-29,32,34,35,37,40,50-53]. This was also the most prevalently measured outcome overall, being reflected in almost all (21/23, 91%) included studies [26-32,34-43,45-54]. Notably, psychosis-related symptoms were consistently reported in conjunction with other outcomes, such as well-being (17/23, 74%) [26-33,36-39,42,43,46-52,54], user experience (15/23, 65%) [27-31,33,34,37-40,43,46-54] and functioning (12/23, 52%) [34,35,37-39,41-43,45,47-49,52,54].

**Figure 2:**
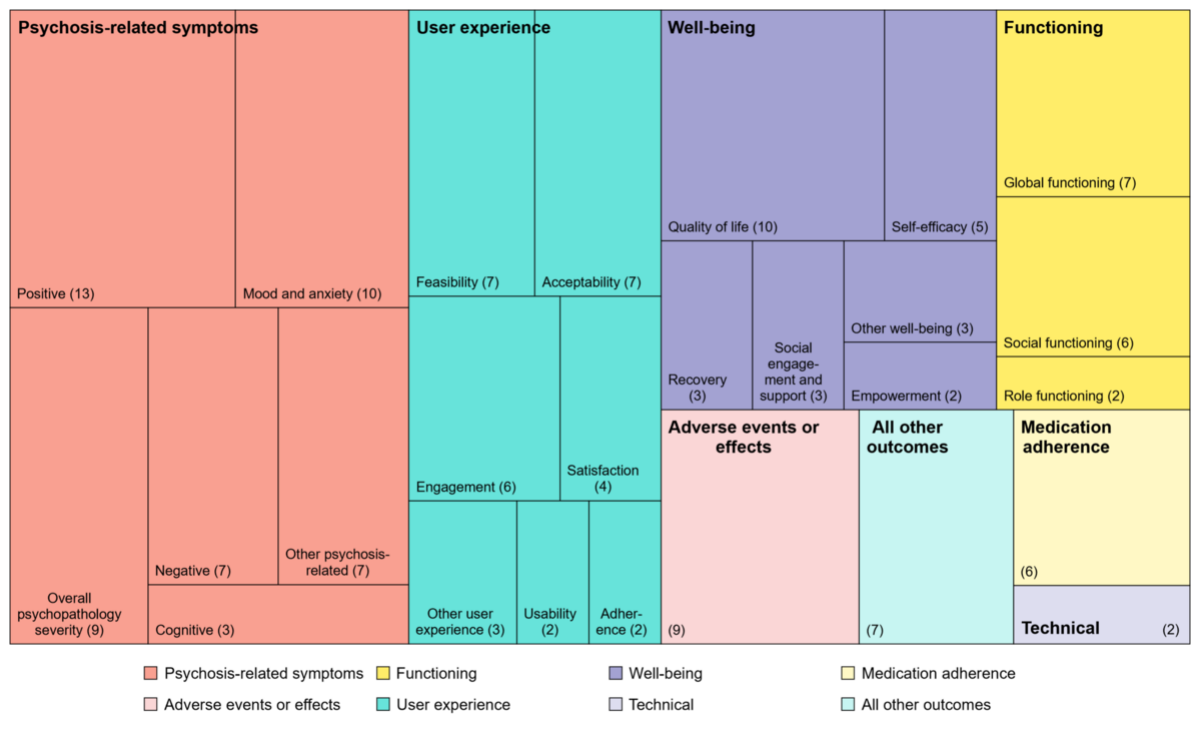
Treemap of reported outcomes in randomized controlled studies of mHealth interventions for psychosis. The size of individual squares is proportional to the frequency of outcome measures.

Figure 2 illustrates the choice of outcome measures and their relative frequencies in included studies. The most frequent measures of psychosis-related symptoms were positive symptoms (13/23, 57%) [26,27,34,35,37-40,42,50-54], mood and anxiety (10/23, 43%) [26,27,34,35,37,38,40,50-54], and overall psychopathology severity (9/23, 39%) [30,31,35,40-43,45,47-51,53]. Less commonly were negative symptoms (7/23, 30%) [34,35,39,40,45,47-49,52,53], other psychosis-related symptoms such as rehospitalization, relapse, and insight (7/23, 30%) [28-32,36,39-41,53], and cognitive symptoms (3/23, 13%) [26,46-49]. Measurement instruments for positive symptoms were highly varied with a mix of observer-rated questionnaires [27-29,35,37,39,40,42,50-54], self-report questionnaires [26,28,29,34,38], and ecological momentary assessments (EMAs) [35] to gauge aspects such as overall positive symptom severity, hallucinations, paranoia, and intensity and distress of psychotic experiences. Similar diversity was observed in measurement instruments for negative symptoms, including overall negative symptom severity [34,35,39,40,53], motivation [47-49,52], and defeatist beliefs [45,52]. On top of self-report questionnaires used to rate mood and anxiety symptoms, EMAs were also leveraged to measure momentary mood [34,35]. Finally, overall psychopathology severity was uniformly observer-rated.

User experience was another commonly measured outcome, with the main domains of interest being feasibility (7/23, 30%) [27,30,31,34,37,40,46,52,53], acceptability (7/23, 30%) [27,30,31,37-39,52,54], and engagement (6/23, 26%) [30,31,38,43,47-51,54]. Feasibility was mostly understood as an objective construct among studies that reported it [27,31,34,37,52,54], with the main measurement instrument being passive data collected from devices. Interpretation of acceptability was slightly more pleomorphic, with most studies using self-report questionnaires to ascertain participants’ views on the intervention [27,30,31,38,39,52,54] and some using objective phone data [37,39] and study retention rates [52]. Of the studies measuring engagement, most studied objective app engagement [30,31,38,40,43,45,50-54], although there were instances of measuring attendance of physical sessions accompanying the mHealth intervention [43,50,51] or subjective service engagement [30,31]. Other measures of user experience include satisfaction, usability, adherence, perceived usefulness, user-friendliness, and negative experiences.

Well-being was uniformly measured through self-report questionnaires. Although quality of life was the most measured construct of well-being (10/23, 43%) [26,28-33,37,43,47-51,54], there was marked diversity in the choice of outcomes within this category, including self-efficacy, recovery, social engagement and support, empowerment, hope, self-esteem, emotional distress, stigma, use of coping strategies and metacognitive beliefs. On the other hand, functioning was mostly measured with observer-rated questionnaires, though other types of measurement instruments were also used [34,35,38,43]. Most instruments measured global (7/23, 30%) [31,35,38,39,41,45,54] and social functioning (6/23, 26%) [34,35,39,42,43,54]. Almost all studies measuring medication adherence used the self-reported Medication Adherence Rating Scale [30,31,33,44,54] except for one study using the Medication Adherence Questionnaire [36] and another collecting data from sensors embedded in medication bottle caps [41]. Contrary to most outcomes, which were compared before and after the intervention, adverse events and technical outcomes were predominantly monitored throughout the study as and when they arose [26-31,37,40,43,45,46,53,54].

**Figure 3:**
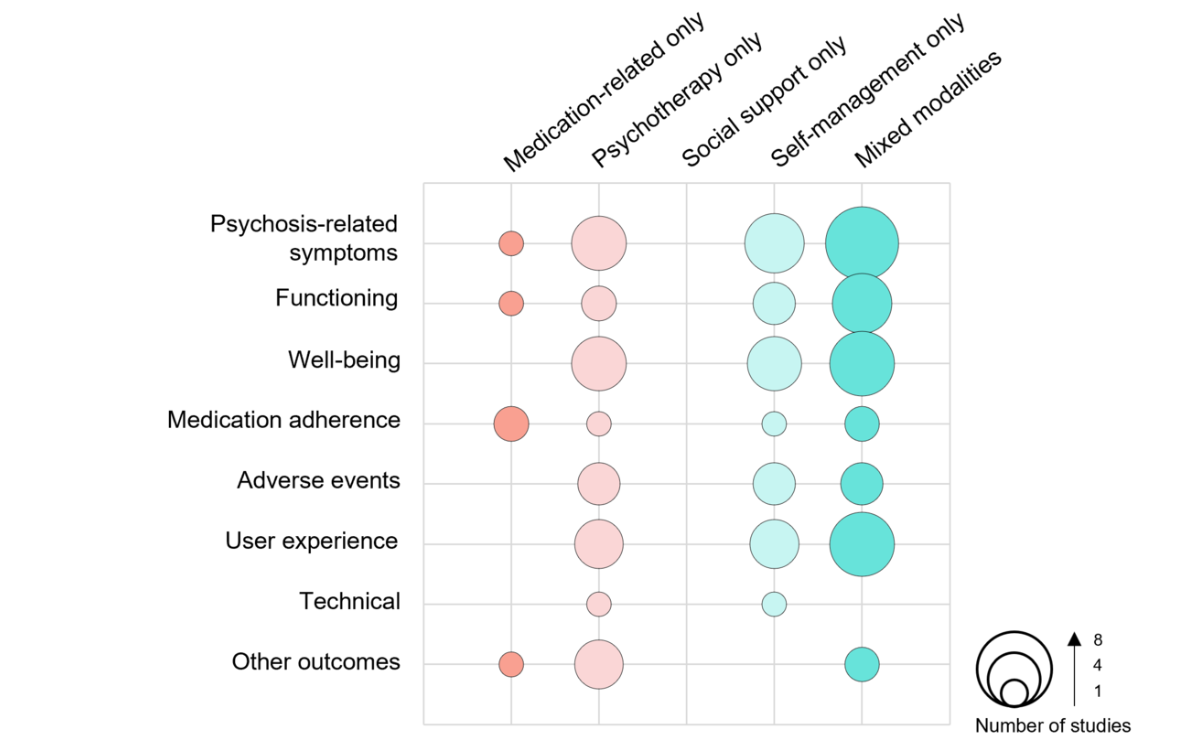
Types of reported outcomes according to the type of mHealth intervention for psychosis.

Choices of outcomes remained multidimensional even after stratifying by the type of intervention studied, as displayed in Figure 3. The severity of psychosis-related symptoms, functioning, and medication adherence were categories of reported outcomes that were common to all intervention types. In contrast, well-being, adverse events, and user experience outcomes were outcome choices common to psychotherapy-only, self-management-only, and mixed modality interventions but not reported in exclusively medication-related mHealth interventions for psychosis. Other outcomes refer to intervention-specific outcomes that cannot be encompassed in the other categories, such as participants’ recall of specific coping heuristics, auditory processing, and motivational incongruence.

## Discussion

### Principal Findings

In this study, we rigorously reviewed the choice of outcomes and measurement instruments in randomized controlled studies evaluating the use of mHealth interventions for adults with psychosis in relation to intervention and control characteristics. The recency of the 23 included studies, particularly the surge in studies published from 2020 onwards, is concordant with the dramatic growth of research in digital mental health in general [12,56], with the COVID-19 pandemic probably being a key catalyst of further development [57,58]. It is encouraging that most mHealth interventions studied are tailored toward outpatient and community care, where sustaining accessibility to mental health services remains a challenge: World Health Organization statistics reveal that almost one-third of countries worldwide still have fewer than 1 psychiatrist in the workforce per 100,000 individuals [59]. The emergence of transdiagnostic mHealth interventions also aligns with a previous systematic review [60], which found a marked increase in studies on mental health applications capable of benefitting multiple groups of patients with different diagnoses. The focus on participants in mid-adulthood may impress upon the possibility of mHealth to manage psychosis in the long term. That being said, younger populations may arguably be a bigger benefactor of mHealth interventions with more pervasive smartphone use and lower engagement with traditional health services [61,62].

The findings of this mapping review strongly suggest that mHealth interventions for adults with psychosis are complex and typically multidimensional in nature. When reviewing the intervention features in isolation, psychotherapy was the most frequently deployed intervention, followed by psychoeducation and active symptom monitoring. Unsurprisingly, CBT was the most common type of psychotherapy delivered, in keeping with its established evidence base and showing its translation toward the mobile realm [63]. Upon further classifying intervention features by domain, most mHealth interventions for psychosis either traversed multiple pillars of Engel’s biopsychosocial model or minimally combined multiple features within the same pillar. Notably, supporting users’ self-management was found to be the most common single-mode intervention. With previous evidence demonstrating how self-management can improve outcomes of people living with severe mental illness [64], this corroborates the role of mHealth interventions in helping users navigate the chronic trajectory of psychosis. Furthermore, we found that the most common medium and channel of delivery were applications and smartphones. This is within expectations given the growing penetration of smartphone usage day-to-day and smartphone uptake among individuals with psychosis [65].

Outcomes reported were similarly numerous, comparable with findings from previous literature [14]. The severity of psychosis-related symptoms—the most reported outcome—was always measured together with other patient-centric measures such as well-being, user experience, or functioning. This bears testament to the transition toward patient-centered care [66] in both the physical and digital spheres and the ensuing importance of evaluating user-related factors [60,67,68] in adopting digital mental health tools. Overall, there needs to be better concordance between measurement instruments of the same outcome categories. We observed multiple studies using mHealth-enabled nascent tools to pick up objective data, such as EMAs [27,30,31,34,35,37,43,50,51] and embedded sensors [41]. This is exciting for refining our understanding of how users’ clinical pictures evolve temporally, but it would require proper validation against traditional measurement instruments [17,69]. Furthermore, included studies vastly differed in the choice and modality of questionnaires, whether observer-rated or self-reported, for variables including hallucinations and social functioning. This might further hinder the reproducibility of results, on top of the myriad of control designs in included studies. Such heterogeneity may directly impact subsequent evaluations of efficacy—for instance, Goldberg et al [12] noted that effect sizes of mHealth-based mental health interventions were smaller when compared with controls with therapeutic intent.

Interestingly, our review dispels the notion that the heterogeneity of outcomes directly reflects intervention diversity—in fact, the spectrum of reported outcomes remained broad despite stratifying by intervention type. This may put into question whether deciding outcomes to be studied based on interventions’ putative mechanisms (like what the ecological interventionist-causal model approach proposes [70]) might still apply when evaluating mHealth interventions. Taken from another perspective, this finding might just be another portrayal of how multifaceted mHealth interventions can be with its integration of multiple components. Their effects may transcend illness severity to simultaneously impact patients’ experiences of their illnesses. In turn, this would strengthen the impetus for holistically assessing clinical and functional outcomes when evaluating mHealth interventions for psychosis. Overall, the plethora of interventions, comparisons, outcomes, and outcome measurement instruments reinforces the need for more high-quality studies with standardized controls and evaluation frameworks to enable validity in further data synthesis. Considering the complexity of between-feature interaction and synergistically affected patient outcomes, the uncertainty still lingers if RCTs are the best evaluation framework for mHealth interventions despite being conventionally perceived as the gold standard of interventional trials.

### Strengths and Limitations

This systematic mapping review has notable strengths. We used a comprehensive search strategy across 5 major bibliographic databases to identify publications reporting on the use of mHealth interventions in adults with psychosis. We have also attempted to minimize missing eligible records by searching the references of included studies. A robust methodology to screen identified articles and perform data extraction in pairs was used to ensure reliable review findings.

There are also some limitations. While including only randomized controlled study designs allowed greater strength in interpreting effects on outcomes, we may not have included nascent interventions in the early stages of development in the process. Furthermore, some potentially relevant studies may have been omitted due to the gamut of terms used in this area.

### Conclusions

mHealth interventions for psychosis should be considered complex interventions probing multiple social and behavioral determinants of health. Randomized controlled studies in the field often report a remarkable breadth of outcomes regardless of the domains they seek to target. Marked variation in the choice of measurement instruments for said outcomes and comparison groups persist amongst studies included in this review. Hence, we recommend a holistic evaluation approach combining clinical and patient-centric outcomes to adequately account for the assortment of mHealth interventions and outcome measures. This would advance our understanding of individuals’ interactions with mHealth interventions that can enhance the person-centered delivery of mental health care for people with psychosis.

## Appendix

Multimedia Appendix 1 PRISMA-ScR (Preferred Reporting Items for Systematic Reviews and Meta-Analyses extension for Scoping Reviews) checklist.

Multimedia Appendix 2 PubMed search strategy.

Multimedia Appendix 3 Characteristics of included studies.

## Abbreviations

CBT: cognitive behavioral therapy
COMET: Core Outcome Measures in Effectiveness Trials
COSMIN: Consensus-based Standards for the selection of health Measurement INstruments
EMA: ecological momentary assessment
iCBT: internet-based cognitive behavioral therapy
ICHOM: International Consortium for Health Outcomes Measurement
mHealth: mobile health
PRISMA-ScR: Preferred Reporting Items for Systematic Reviews and Meta-Analyses extension for Scoping Reviews
RCT: randomized controlled trial
YLDs: years lived with disability

## Data Availability

All data generated or analyzed during this study are included in this published article and its supplementary information files.

## References

[1] American Psychiatric Association (2013). Diagnostic and Statistical Manual of Mental Disorders.

[2] GBD 2019 Mental Disorders Collaborators (2022). Global, regional, and national burden of 12 mental disorders in 204 countries and territories, 1990-2019: a systematic analysis for the global burden of disease study 2019. Lancet Psychiatry.

[3] Tandon R, Nasrallah HA, Keshavan MS (2009). Schizophrenia, "just the facts" 4. Clinical features and conceptualization. Schizophr Res.

[4] Whiteford H, Ferrari A, Degenhardt L (2016). Global burden Of disease studies: implications for mental and substance use disorders. Health Aff (Millwood).

[5] Kadakia A, Fan A, Marden J, Dembek C, Catillon M, Anderson A, Williams R (2022). The economic burden of schizophrenia in the United States in 2019. CNS Spectr.

[6] World Health Organization (2016). mHealth: use of mobile wireless on public health. Exec Board 139th Session.

[7] Firth J, Cotter J, Torous J, Bucci S, Firth JA, Yung AR (2016). Mobile phone ownership and endorsement of "mHealth" Among people with psychosis: a meta-analysis of cross-sectional studies. Schizophr Bull.

[8] Ahmed I, Ahmad NS, Ali S, Ali S, George A, Saleem Danish H, Uppal E, Soo J, Mobasheri MH, King D, Cox B, Darzi A (2018). Medication adherence apps: review and content analysis. JMIR Mhealth Uhealth.

[9] Rathbone AL, Clarry L, Prescott J (2017). Assessing the efficacy of mobile health apps using the basic principles of cognitive behavioral therapy: systematic review. J Med Internet Res.

[10] Achtyes ED, Gega L, Linnaranta O (2021). Editorial: mHealth: self-management and complementary psychiatric treatment. Front Psychiatry.

[11] Marcolino MS, Oliveira JAQ, D'Agostino M, Ribeiro AL, Alkmim MBM, Novillo-Ortiz D (2018). The impact of mHealth interventions: systematic review of systematic reviews. JMIR Mhealth Uhealth.

[12] Goldberg SB, Lam SU, Simonsson O, Torous J, Sun S (2022). Mobile phone-based interventions for mental health: a systematic meta-review of 14 meta-analyses of randomized controlled trials. PLOS Digit Health.

[13] Chivilgina O, Wangmo T, Elger BS, Heinrich T, Jotterand F (2020). mHealth for schizophrenia spectrum disorders management: a systematic review. Int J Soc Psychiatry.

[14] Gire N, Farooq S, Naeem F, Duxbury J, McKeown M, Kundi PS, Chaudhry IB, Husain N (2017). mHealth based interventions for the assessment and treatment of psychotic disorders: a systematic review. Mhealth.

[15] Camacho E, Levin L, Torous J (2019). Smartphone apps to support coordinated specialty care for prodromal and early course schizophrenia disorders: systematic review. J Med Internet Res.

[16] Alvarez-Jimenez M, Alcazar-Corcoles MA, González-Blanch C, Bendall S, McGorry PD, Gleeson JF (2014). Online, social media and mobile technologies for psychosis treatment: a systematic review on novel user-led interventions. Schizophr Res.

[17] Firth J, Torous J (2015). Smartphone apps for schizophrenia: a aystematic review. JMIR Mhealth Uhealth.

[18] Clarke S, Hanna D, Mulholland C, Shannon C, Urquhart C (2019). A systematic review and meta-analysis of digital health technologies effects on psychotic symptoms in adults with psychosis. Psychosis Routledge.

[19] James KL, Randall NP, Haddaway NR (2016). A methodology for systematic mapping in environmental sciences. Environ Evid.

[20] Loh PY (2023). OSF Registry. mHealth Interventions for Adult Patients With Psychosis: A Systematic Mapping Review.

[21] Page MJ, McKenzie JE, Bossuyt PM, Boutron I, Hoffmann TC, Mulrow CD, Shamseer L, Tetzlaff JM, Akl EA, Brennan SE, Chou R, Glanville J, Grimshaw JM, Hróbjartsson A, Lalu MM, Li T, Loder EW, Mayo-Wilson E, McDonald S, McGuinness LA, Stewart LA, Thomas J, Tricco AC, Welch VA, Whiting P, Moher D (2021). The PRISMA 2020 statement: an updated guideline for reporting systematic reviews. BMJ.

[22] Lattie EG, Stiles-Shields C, Graham AK (2022). An overview of and recommendations for more accessible digital mental health services. Nat Rev Psychol.

[23] McKenzie E, Matkin L, Sousa Fialho L, Emelurumonye IN, Gintner T, Ilesanmi C, Jagger B, Quinney S, Anderson E, Baandrup L, Bakhshy AK, Brabban A, Coombs T, Correll CU, Cupitt C, Keetharuth AD, Lima DN, McCrone P, Moller M, Mulder CL, Roe D, Sara G, Shokraneh F, Sin J, Woodberry KA, Addington D, Psychotic Disorders Working Group of the International Consortium for Health Outcomes Measurement (2022). Developing an international standard set of patient-reported outcome measures for psychotic disorders. Psychiatr Serv.

[24] Dodd S, Clarke M, Becker L, Mavergames C, Fish R, Williamson PR (2018). A taxonomy has been developed for outcomes in medical research to help improve knowledge discovery. J Clin Epidemiol.

[25] Prinsen CAC, Vohra S, Rose MR, Boers M, Tugwell P, Clarke M, Williamson PR, Terwee CB (2016). How to select outcome measurement instruments for outcomes included in a "Core Outcome Set" - a practical guideline. Trials.

[26] Garety P, Ward T, Emsley R, Greenwood K, Freeman D, Fowler D, Kuipers E, Bebbington P, Rus-Calafell M, McGourty A, Sacadura C, Collett N, James K, Hardy A (2021). Effects of SlowMo, a blended digital therapy targeting reasoning, on paranoia among people with psychosis: a randomized clinical trial. JAMA Psychiatry.

[27] Bell IH, Rossell SL, Farhall J, Hayward M, Lim MH, Fielding-Smith SF, Thomas N (2020). Pilot randomised controlled trial of a brief coping-focused intervention for hearing voices blended with smartphone-based ecological momentary assessment and intervention (SAVVy): Feasibility, acceptability and preliminary clinical outcomes. Schizophr Res.

[28] Westermann S, Rüegg N, Lüdtke T, Moritz S, Berger T (2020). Internet-based self-help for psychosis: findings from a randomized controlled trial. J Consult Clin Psychol.

[29] Lüdtke T, Platow-Kohlschein H, Rüegg N, Berger T, Moritz S, Westermann S (2020). Mindfulness mediates the effect of a psychological online intervention for psychosis on self-reported hallucinations: a secondary analysis of voice hearers from the EviBaS trial. Front Psychiatry.

[30] Gumley AI, Bradstreet S, Ainsworth J, Allan S, Alvarez-Jimenez M, Aucott L, Birchwood M, Briggs A, Bucci S, Cotton SM, Engel L, French P, Lederman R, Lewis S, Machin M, MacLennan G, McLeod H, McMeekin N, Mihalopoulos C, Morton E, Norrie J, Schwannauer M, Singh SP, Sundram S, Thompson A, Williams C, Yung AR, Farhall J, Gleeson J (2022). The EMPOWER blended digital intervention for relapse prevention in schizophrenia: a feasibility cluster randomised controlled trial in scotland and Australia. Lancet Psychiatry.

[31] Gumley AI, Bradstreet S, Ainsworth J, Allan S, Alvarez-Jimenez M, Birchwood M, Briggs A, Bucci S, Cotton S, Engel L, French P, Lederman R, Lewis S, Machin M, MacLennan G, McLeod H, McMeekin N, Mihalopoulos C, Morton E, Norrie J, Reilly F, Schwannauer M, Singh SP, Sundram S, Thompson A, Williams C, Yung A, Aucott L, Farhall J, Gleeson J (2022). Digital smartphone intervention to recognise and manage early warning signs in schizophrenia to prevent relapse: the EMPOWER feasibility cluster RCT. Health Technol Assess.

[32] Homan P, Schooler NR, Brunette MF, Rotondi A, Ben-Zeev D, Gottlieb JD, Mueser KT, Achtyes ED, Gingerich S, Marcy P, Meyer-Kalos P, Hauser M, John M, Robinson DG, Kane JM (2022). Relapse prevention through health technology program reduces hospitalization in schizophrenia. Psychol. Med.

[33] Röhricht F, Padmanabhan R, Binfield P, Mavji D, Barlow S (2021). Simple mobile technology health management tool for people with severe mental illness: a randomised controlled feasibility trial. BMC Psychiatry.

[34] Hanssen E, Balvert S, Oorschot M, Borkelmans K, van Os J, Delespaul P, Fett AK (2020). An ecological momentary intervention incorporating personalised feedback to improve symptoms and social functioning in schizophrenia spectrum disorders. Psychiatry Res.

[35] Myin-Germeys I, van Aubel E, Vaessen T, Steinhart H, Klippel A, Lafit G, Viechtbauer W, Batink T, van Winkel R, van der Gaag M, van Amelsvoort T, Marcelis M, Schirmbeck F, de Haan L, Reininghaus U (2022). Efficacy of acceptance and commitment therapy in daily life in early psychosis: results from the multi-center INTERACT randomized controlled trial. Psychother Psychosom.

[36] Zhu X, Li M, Liu P, Chang R, Wang Q, Liu J (2020). A mobile health application-based strategy for enhancing adherence to antipsychotic medication in schizophrenia. Arch Psychiatr Nurs.

[37] Lewis S, Ainsworth J, Sanders C, Stockton-Powdrell C, Machin M, Whelan P, Hopkins R, He Z, Applegate E, Drake R, Bamford C, Roberts C, Wykes T (2020). Smartphone-enhanced symptom management in psychosis: open, randomized controlled trial. J Med Internet Res.

[38] Ben-Zeev D, Chander A, Tauscher J, Buck B, Nepal S, Campbell A, Doron G (2021). A smartphone intervention for people with serious mental illness: fully remote randomized controlled trial of CORE. J Med Internet Res.

[39] Vitger T, Hjorthøj C, Austin SF, Petersen L, Tønder ES, Nordentoft M, Korsbek L (2022). A smartphone app to promote patient activation and support shared decision-making in people with a diagnosis of schizophrenia in outpatient treatment settings (Momentum Trial): randomized controlled assessor-blinded trial. J Med Internet Res.

[40] Krzystanek M, Krysta K, Borkowski M, Skałacka K, Przybyło J, Pałasz A, Mucic D, Martyniak E, Waszkiewicz N (2020). The effect of smartphone-based cognitive training on the functional/cognitive markers of schizophrenia: a one-year randomized study. J Clin Med.

[41] Tessier A, Dupuy M, Baylé FJ, Herse C, Lange A, Vrijens B, Schweitzer P, Swendsen J, Misdrahi D (2020). Brief interventions for improving adherence in schizophrenia: a pilot study using electronic medication event monitoring. Psychiatry Res.

[42] Han M, Lee K, Kim M, Heo Y, Choi H (2023). Effects of a metacognitive smartphone intervention with weekly mentoring sessions for individuals with schizophrenia: a quasi-experimental study. J Psychosoc Nurs Ment Health Serv.

[43] Dabit S, Quraishi S, Jordan J, Biagianti B (2021). Improving social functioning in people with schizophrenia-spectrum disorders via mobile experimental interventions: results from the CLIMB pilot trial. Schizophr Res Cogn.

[44] Lisinge E (2020). Improving medication adherence in patients diagnosed with schizophrenia using cell phone apps in addition to attending focus group therapy. Diss Abstr Int Sect B Sci Eng.

[45] Depp CA, Perivoliotis D, Holden J, Dorr J, Granholm EL (2019). Single-session mobile-augmented intervention in serious mental illness: a three-arm randomized controlled trial. Schizophr Bull.

[46] Roberts DL, Liu PYT, Busanet H, Maples N, Velligan D (2017). A tablet-based intervention to manipulate social cognitive bias in schizophrenia. American Journal of Psychiatric Rehabilitation.

[47] Biagianti B, Fisher M, Howard L, Rowlands A, Vinogradov S, Woolley J (2017). Feasibility and preliminary efficacy of remotely delivering cognitive training to people with schizophrenia using tablets. Schizophr Res Cogn.

[48] Fisher M, Nahum M, Howard E, Rowlands A, Brandrett B, Kermott A, Woolley J, Vinogradov S (2017). Supplementing intensive targeted computerized cognitive training with social cognitive exercises for people with schizophrenia: an interim report. Psychiatr Rehabil J.

[49] Miley K, Fisher M, Nahum M, Howard E, Rowlands A, Brandrett B, Woolley J, Hooker CI, Biagianti B, Ramsay I, Vinogradov S (2020). Six month durability of targeted cognitive training supplemented with social cognition exercises in schizophrenia. Schizophr Res Cogn.

[50] Ben-Zeev D, Brian RM, Jonathan G, Razzano L, Pashka N, Carpenter-Song E, Drake RE, Scherer EA (2018). Mobile health (mHealth) versus clinic-based group intervention for people with serious mental illness: a randomized controlled trial. Psychiatr Serv.

[51] Ben-Zeev D, Buck B, Chu PV, Razzano L, Pashka N, Hallgren KA (2019). Transdiagnostic mobile health: smartphone intervention reduces depressive symptoms in people with mood and psychotic disorders. JMIR Ment Health.

[52] Schlosser DA, Campellone TR, Truong B, Etter K, Vergani S, Komaiko K, Vinogradov S (2018). Efficacy of PRIME, a mobile app intervention designed to improve motivation in young people with schizophrenia. Schizophr Bull.

[53] Krzystanek M, Borkowski M, Skałacka K, Krysta K (2019). A telemedicine platform to improve clinical parameters in paranoid schizophrenia patients: results of a one-year randomized study. Schizophr Res.

[54] Bucci S, Barrowclough C, Ainsworth J, Machin M, Morris R, Berry K, Emsley R, Lewis S, Edge D, Buchan I, Haddock G (2018). Actissist: proof-of-concept trial of a theory-driven digital intervention for psychosis. Schizophr Bull.

[55] Engel GL (1977). The need for a new medical model: a challenge for biomedicine. Science.

[56] Drissi N, Ouhbi S, Janati Idrissi MA, Fernandez-Luque L, Ghogho M (2020). Connected mental health: systematic mapping study. J Med Internet Res.

[57] Philippe TJ, Sikder N, Jackson A, Koblanski ME, Liow E, Pilarinos A, Vasarhelyi K (2022). Digital health interventions for delivery of mental health care: systematic and comprehensive meta-review. JMIR Ment Health.

[58] Baños RM, Herrero R, Vara MD (2022). What is the current and future status of digital mental health interventions?. Span. J. Psychol.

[59] World Health Organization (2019). Mental Health Workforce Statistics.

[60] Miralles I, Granell C, Díaz-Sanahuja L, Van Woensel W, Bretón-López J, Mira A, Castilla D, Casteleyn S (2020). Smartphone apps for the treatment of mental disorders: systematic review. JMIR Mhealth Uhealth.

[61] Burns J, Birrell E (2014). Enhancing early engagement with mental health services by young people. Psychol Res Behav Manag.

[62] Rus-Calafell M, Schneider S (2020). Are we there yet?!-a literature review of recent digital technology advances for the treatment of early psychosis. Mhealth.

[63] Kumar V, Sattar Y, Bseiso A, Khan S, Rutkofsky IH (2017). The effectiveness of internet-based cognitive behavioral therapy in treatment of psychiatric disorders. Cureus.

[64] Lean M, Fornells-Ambrojo M, Milton A, Lloyd-Evans B, Harrison-Stewart B, Yesufu-Udechuku A, Kendall T, Johnson S (2019). Self-management interventions for people with severe mental illness: systematic review and meta-analysis. Br J Psychiatry.

[65] Buck B, Chander A, Tauscher J, Nguyen T, Monroe-DeVita M, Ben-Zeev D (2021). mHealth for young adults with early psychosis: user preferences and their relationship to attitudes about treatment-seeking. J Technol Behav Sci.

[66] Ellen ME, Shach R, Balicer RD (2018). Helping patients help themselves: supporting the healthcare journey. Patient Educ Couns.

[67] Vial S, Boudhraâ S, Dumont M (2022). Human-centered design approaches in digital mental health interventions: exploratory mapping review. JMIR Ment Health.

[68] Kaveladze BT, Wasil AR, Bunyi JB, Ramirez V, Schueller SM (2022). User experience, engagement, and popularity in mental health apps: secondary analysis of app analytics and expert app reviews. JMIR Hum Factors.

[69] Kirkpatrick B, Luther L, Strauss GP (2023). Negative symptoms in the clinic: we treat what we can describe. Br J Psychiatry.

[70] Reininghaus U, Depp CA, Myin-Germeys I (2016). Ecological interventionist causal models in psychosis: targeting psychological mechanisms in daily life. Schizophr Bull.

