# Supplementary material for: Characteristics and Outcomes of mHealth Interventions in Psychosis: Systematic Mapping Review
Source: J Med Internet Res. 2024 Dec 23;26:e55924. doi: 10.2196/55924 (PMC11704647; doi:10.2196/55924)
Supplement: Multimedia Appendix 2 [file jmir_v26i1e55924_app2.docx]

## mHealth Interventions for Adults with Psychosis: A Systematic Mapping Review

#### Multimedia Appendix 2. PubMed search strategy

#1. digital technology[MeSH Terms]

#2. mobile applications[MeSH Terms]

#3. smartphone[MeSH Terms]

#4. (digital technology [Title/Abstract]) OR (digital therapeutics [Title/Abstract]) OR (DTx [Title/Abstract]) OR (mobile application* [Title/Abstract]) OR (app* [Title/Abstract]) OR (eHealth [Title/Abstract]) OR (e-health [Title/Abstract]) OR (mHealth [Title/Abstract]) OR (m-Health [Title/Abstract]) OR (mobile health [Title/Abstract]) OR (smartphone* [Title/Abstract]) OR (digital health [Title/Abstract])

#5. #1 OR #2 OR #3 OR #4

#6. Schizophrenia Spectrum and Other Psychotic Disorders [MeSH Terms]

#7. Schizophrenia [MeSH Terms]

#8. Psychotic Disorders [MeSH Terms]

#9. Affective Disorders, Psychotic [MeSH Terms]

#10. (Schizophrenia [Title/Abstract]) OR (Psychotic Disorder* [Title/Abstract]) OR (Psychosis [Title/Abstract])

#11. #6 OR #7 OR #8 OR #9 OR #10

#12. #5 AND #11
