# Supplementary material for: Characteristics and Outcomes of mHealth Interventions in Psychosis: Systematic Mapping Review
Source: J Med Internet Res. 2024 Dec 23;26:e55924. doi: 10.2196/55924 (PMC11704647; doi:10.2196/55924)
Supplement: Multimedia Appendix 3 [file jmir_v26i1e55924_app3.docx]

**Multimedia Appendix 3**: Characteristics of included studies

| Study ID [Ref] | Country | Study design | Number of participants | Target disorder | Intervention name | Intervention features | Delivery format of intervention | Comparison | Outcome categories | Type of measurement instruments |
| --- | --- | --- | --- | --- | --- | --- | --- | --- | --- | --- |
| Roberts 2017 [45] | USA | RCT | 24 | Psychotic disorders + mood disorders | MEBi-Self | Psychotherapy | Self-guided | Different version of mHealth intervention | Psychosis-specific symptoms, well-being, user experience, technical | Observer-rated questionnaire, self-report questionnaire, mobile device data |
| Biagianti 2017 [46]; Fisher 2017 [47]; Miley 2019 [48] | USA | RCT | 111 | Psychotic disorders only | NA | Psychotherapy | Not specified | Different version of mHealth intervention | Psychosis-specific symptoms, functioning, well-being, user experience | Observer-rated questionnaire, self-report questionnaire |
| Garety 2021 [26] | UK | RCT | 362 | Psychotic disorders only | SlowMo | Psychotherapy | Blended | Treatment as usual | Psychosis-specific symptoms, well-being, adverse events | Observer-rated questionnaire, self-report questionnaire, mobile device data |
| Bell 2020 [27] | Australia | RCT | 34 | Psychotic disorders + mood disorders | SAVVy | Active self-monitoring, personalized coping recommendations | Blended | Treatment as usual | Psychosis-specific symptoms, well-being, adverse events, user experience | Observer-rated questionnaire, self-report questionnaire |
| Westermann 2020 [28]; Lüdtke 2020 [29] | Switzerland, Germany | RCT | 101 | Psychotic disorders only | EviBaS | Psychotherapy | Guided | Waitlist control | Psychosis-specific symptoms, well-being, adverse events, user experience | Observer-rated questionnaire, self-report questionnaire |
| Gumley 2022-1 [30]; Gumley 2022-2 [31] | Australia, UK | Cluster RCT | 73 | Psychotic disorders only | EMPOWER | Active self-monitoring, personalized coping recommendations | Guided | Treatment as usual | Psychosis-specific symptoms, well-being, medication adherence, adverse events, user experience, technical | Observer-rated questionnaire, self-report questionnaire, mobile device data |
| Homan 2022 [32] | USA | Quasi-RCT | 438 | Psychotic disorders only | Health Technology Program | Psychotherapy, psychoeducation, peer support | Blended | Treatment as usual | Psychosis-specific symptoms, well-being | Patient case records |
| Rohricht 2021 [33] | UK | RCT | 65 | Psychotic disorders + mood disorders | Florence | Emergency assistance, medication adherence, active self-monitoring, personalized coping recommendations | Blended | Treatment as usual | Well-being, medication adherence, user experience | Patient interviews, self-report questionnaire, mobile device data |
| Hanssen 2020 [34] | The Netherlands | RCT | 50 | Psychotic disorders only | SMARTapp | Medication adherence, active self-monitoring, personalized coping recommendations | Self-guided | Another mHealth intervention | Psychosis-specific symptoms, functioning, user experience | Self-report questionnaire, EMA, mobile device data |
| Ben-Zeev 2018 [49]; Ben-Zeev 2019[50] | USA | RCT | 163 | Psychotic disorders + mood disorders | FOCUS | Psychoeducation, active self-monitoring | Guided | Non-mHealth intervention | Psychosis-specific symptoms, well-being, user experience | Observer-rated questionnaire, self-report questionnaire, mobile device data, session attendance |
| Myin-Germeys 2022 [35] | The Netherlands, Belgium | RCT | 148 | Ultra-High Risk + psychotic disorders | ACT-DL | Active self-monitoring, personalized coping recommendations | Blended | Treatment as usual | Psychosis-specific symptoms, functioning | Observer-rated questionnaire, self-report questionnaire, EMA |
| Zhu 2020 [36] | China | RCT | 84 | Psychotic disorders only | NA | Psychoeducation, medication adherence | Guided | Treatment as usual | Psychosis-specific symptoms, well-being, medication adherence | Observer-rated questionnaire, self-report questionnaire |
| Lewis 2020 [37] | UK | RCT | 81 | Not specified | ClinTouch | Active self-monitoring | Guided | Treatment as usual | Psychosis-specific symptoms, functioning, well-being, adverse events, user experience | Observer-rated questionnaire, self-report questionnaire, patient interviews, mobile device data |
| Schlosser 2018 [51] | USA | RCT | 43 | Psychotic disorders only | PRIME | Psychotherapy, psychoeducation, peer support, behavioural activation, mindfulness | Guided | Waitlist control | Psychosis-specific symptoms, functioning, well-being, user experience | Observer-rated questionnaire, self-report questionnaire, mobile device data |
| Ben-Zeev 2021 [38] | USA | RCT | 315 | Psychotic disorders + mood disorders | CORE | Psychotherapy, psychoeducation | Not specified | Waitlist control | Psychosis-specific symptoms, functioning, well-being, user experience | Self-report questionnaire, mobile device data |
| Vitger 2022 [39] | Denmark | RCT | 194 | Psychotic disorders only | NA | Shared decision making | Blended | Treatment as usual | Psychosis-specific symptoms, functioning, well-being, user experience | Observer-rated questionnaire, self-report questionnaire, patient data register, mobile device data |
| Krzystanek 2019 [53]; Krzystanek 2020 [40] | Poland | RCT | 290 | Psychotic disorders only | MONEO Platform | Psychotherapy, psychoeducation, medication adherence, teleconsultations | Blended | Placebo | Psychosis-specific symptoms, adverse events, user experience | Observer-rated questionnaire, MONEO platform data |
| Bucci 2018 [54] | UK | RCT | 36 | Not specified | Actissist | Psychotherapy | Self-guided | Another mHealth intervention | Psychosis-specific symptoms, functioning, well-being, medication adherence, adverse events, user experience | Observer-rated questionnaire, self-report questionnaire, patient interviews, mobile device data |
| Tessier 2020 [41] | France | RCT | 33 | Not specified | MEMS | Medication adherence | Self-guided | TAU + non-mHealth intervention | Psychosis-specific symptoms, functioning, medication adherence | Observer-rated questionnaire, sensor data from MEMS |
| Depp 2019 [52] | USA | RCT | 255 | Psychotic disorders + mood disorders | CBT2go | Psychotherapy, psychoeducation, medication adherence | Blended | TAU + another mHealth intervention | Psychosis-specific symptoms, functioning, adverse events | Observer-rated questionnaire, self-report questionnaire |
| Han 2022 [42] | Korea | Quasi-RCT | 44 | Psychotic disorders only | MCI-S | Psychoeducation | Blended | Different version of mHealth intervention | Psychosis-specific symptoms, functioning, well-being | Observer-rated questionnaire, self-report questionnaire |
| Dabit 2021 [43] | USA / Canada / France / India/ UK | RCT | 30 | Psychotic disorders only | CLIMB | Psychotherapy, active self-monitoring, peer support | Blended | Another mHealth intervention | Psychosis-specific symptoms, functioning, well-being, adverse events, user experience | Observer-rated questionnaire, self-report questionnaire, mobile device data, session attendance |
| Lisinge 2020 [44] | USA | RCT | 30 | Psychotic disorders only | Mymedschedule Plus | Medication adherence | Blended | Non-mHealth intervention | Medication adherence | Self-report questionnaire |

**EMA**: ecological momentary assessment; **RCT**: randomized controlled trial; **TAU**: treatment as usual
